# COVID-19 vaccine adoption and hesitancy among older Veterans

**DOI:** 10.1186/s12889-022-13882-7

**Published:** 2022-08-11

**Authors:** Tamar Wyte-Lake, Cari Levy, Sona Hovsepian, Yvonne Mudoh, Cheryl Schmitz, Aram Dobalian

**Affiliations:** 1 Veterans Emergency Management Evaluation Center (VEMEC), Office of Patient Care Services (Population Health), US Department of Veterans Affairs, Veterans Health Administration, 16111 Plummer St. MS-152, Bldg 22, Rm 113, North Hills, CA 91343 USA; 2 Department of Family Medicine, Oregon Health & Science University, Portland, OR USA; 3 US Department of Veterans Affairs, Rocky Mountain Regional Veterans Affairs Medical Center, Aurora, CO USA; 4 Denver School of Medicine, University of Colorado, Aurora, OR USA; 5 Veterans Emergency Management Evaluation Center, Office of Patient Care Services (Population Health), US Department of Veterans Affairs, Veterans Health Administration, North Hills, CA USA; 6 US Department of Veterans Affairs, Office of Geriatrics and Extended Care, Washington, DC USA; 7 Division of Health Services Management and Policy, The Ohio State University College of Public Health, Columbus, OH USA

**Keywords:** COVID-19 Vaccination, Geriatric Primary Care, Older old, Vaccine hesitancy, Vaccination barriers

## Abstract

**Background:**

Older adults are particularly at risk for severe illness or death from COVID-19. Accordingly, the Veterans Health Administration (VA) has prioritized this population group in its COVID-19 vaccination strategy. This study examines the receptivity of Veterans enrolled in the VA’s Geriatric Patient Aligned Care Team (GeriPACT) to receiving the COVID-19 vaccine. GeriPACT is an outpatient primary care program that utilizes multi-disciplinary teams to provide health services to older Veterans.

**Methods:**

We conducted semistructured interviews with 42 GeriPACT-enrolled Veterans from five states. Participants were asked to identify barriers to vaccine acceptance. We gathered data from January-March 2021 and analyzed them using qualitative methods.

**Results:**

Both White and African American GeriPACT Veterans had minimal vaccine hesitancy towards the COVID-19 vaccine. On-line registration and ineligibility of a spouse/caregiver for vaccination were primary barriers to early vaccination.

**Conclusions:**

As the first wave of early adopters of the COVID-19 vaccination effort nears completion, targeted strategies are needed to understand and respond to vaccine hesitancy to lower the risk of subsequent waves of infections. The 2021 SAVE LIVES Act, begins to address identified vaccination barriers by permitting vaccination of Veteran spouses and caregivers, but consideration must be given to creating alternatives to on-line registration and allowing spouses and caregivers to register for appointments together.

## Background

COVID-19 vaccine eligibility is now open to all persons aged 16 or older in the United States. Having surmounted the initial hurdles of COVID-19 vaccine development and distribution, addressing and decreasing vaccine hesitancy is the next essential step to achieve herd immunity and return to a new “normal.”

Older adults, particularly those with chronic conditions, fall into the most vulnerable categories for all phases of COVID-19, from contracting the virus to risk of becoming critically ill or dying [1]. Furthermore, their vulnerability to COVID-19 is exacerbated in many instances by reliance on a caregiver who goes in and out of the home, and who thereby increases their risk for contracting COVID-19 from the community. Little is known about COVID-19 specific vaccination acceptance among older adults, particularly within communities of color that have historically demonstrated lower vaccine acceptance. The extant literature suggests vaccine hesitancy relates to distrust of authorities, limited access to high-quality sources of information, frequent lack of connection to a regular source of health, and challenges engaging with online registration systems [2–5]. As such, some older adults, particularly communities of color, [6] may be more hesitant to become vaccinated.

The Veterans Health Administration’s (VA) Geriatric Patient Aligned Care Team (GeriPACT) program, has about 60,000 enrolled Veterans and is akin to a primary care medical home (PCMH) model. It is structured as an inter-disciplinary, team-based, primary care team that provides a higher level of support than an average Primary Care PACT team. GeriPACT patients are older than traditional PACT patients and have high prevalence of functional decline, chronic comorbidities, and memory loss [7]. Due to their high-risk for COVID-19, GeriPACT-enrolled Veterans were within the first groups targeted by VA for vaccine distribution. GeriPACT-enrolled Veterans, who are at risk of lower vaccine uptake because they are often socially isolated, may fear leaving the home, [8] and include multiple population groups with historically high vaccine hesitancy, including African Americans and people of lower educational attainment [9, 10].

Peretti-Watel, Larson [3] describe a theoretical framework that considers vaccine hesitancy to be a decision-making process that depends on several factors, including confidence in health authorities. They propose that vaccine hesitancy is an intermediate position on a continuum, and that individuals’ attitudes and beliefs can move along that continuum based on external factors such as influence of trusted advisors.

Using Peretti-Watel’s framework as a guide, this study explores receptivity of Veterans enrolled in the VA’s GeriPACT program to the COVID-19 vaccine. Three factors were explored in detail; (1) attitudes and beliefs of Veterans about COVID-19 vaccination; (2) how Veterans engaged with their healthcare team around vaccination; and (3) the impact of these and other factors on their likelihood of being vaccinated.

## Methods

This study employed a qualitative study design using semi-structured telephone interviews with Veterans enrolled in the VA’s GeriPACT program. The interview guide was created with a specific focus on external factors that could alter an individual’s attitudes and beliefs about vaccination, including the identification of trusted sources within the VA healthcare system and barriers to vaccine acceptance. This study was given approval by the Institutional Review Board of the Greater Los Angeles VA Medical Center.

### Sample

The study was initially presented by project team members on the January 2021 national GeriPACT monthly meeting, which is attended nationally by GeriPACT practitioners and staff to discuss current events. Recruitment was initiated by asking practitioners from the GeriPACT programs to provide a list of up to 10 names of Veterans enrolled in their program. The only inclusion criterion was that patients were enrolled in GeriPACT, although we did inform programs that we wished to include a mix of both vaccinated and unvaccinated patients. There were not exclusion criteria presented. Practitioners from five sites agreed to participate, and patients were chosen from their patient panels.

### Data collection methods

A total of five GeriPACT programs from five states (Arkansas, Colorado, Michigan, Missouri, California) participated in the study. The research team received 64 names from GeriPACT programs. Project staff then independently consented the Veterans. Of the 64 names received, researchers spoke with 42 Veterans enrolled in the GeriPACT programs (64% White, 29% African American, and 7% Other). The remaining 22 Veterans either declined to be interviewed or were unable to be contacted. The first 11 Veterans who agreed to participate in an interview (4 White and 7 African American) participated in individual, in-depth interviews to understand their vaccine decision-making process. After each interview, the project team discussed the major themes presented. After the eleventh interview, the project team decided that saturation was reached, as no new themes were being introduced. In order to receive validation about COVID-19 vaccine uptake from a larger sample, brief interviews continued with the remaining 31 Veterans, verifying whether they had already received the COVID-19 vaccine, or intended to receive it when eligible (See Tables 1 and 2).

**Table 1:** GeriPACT Veterans recruitment details

	**White**	**African American**	**Other**
Interviewed	27	12	3
Left message/unable to reach	12	6	0
Declined to speak with us	1	1	0
**Received COVID-19 vaccine anywhere**	Yes: 20 No: 1 Waiting for appt: 6	Yes: 11 No: 1 Waiting for appt: 0	Yes: 3 No: 0 Waiting for appt: 0
**Received COVID-19 vaccine from VA**	Yes: 17 (85%) No: 3	Yes: 9 (82%) No: 5	Yes: 2 (66%) No: 1
**State of Residence**			
** Michigan**	5		1
** Colorado**	5		
** California**		5	
** Arkansas**	11	2	
** Missouri**	6	5	2

**Table 2:** Demographic characteristics of in-depth interviewed participants (*N* = 11)

	**White (*n* = 4)**	**African American (*n* = 7)**	**Total**
**Age**			
Mean	81	70	74
Range	71–95 Years	74–91 Years	71–95
**Living Status**			
Alone/Residential Facility		1	1
With Spouse/Significant other	4	3	7
With Caregiver/Relative		3	3
**Years Receiving Care at VA**			
10–20		1	1
20 +	4	6	10
Years Receiving Care in GeriPACT program			
< 2 years	1	1	2
≥ 2 years	3	6	9

Interviews were conducted from January 27-March 17, 2021, with 92% of interviews completed by February 27^th^. The project team refined the guide as recruitment progressed, probing around issues that emerged as relevant from prior interviews. The interview guide queried respondents about: 1) whether Veterans had received the COVID-19 vaccine, 2) where they received or expected to receive it, 3) experience with and trust in their VA healthcare team, 4) sources of information about COVID-19 and the vaccine, 5) social support, and 6) concerns about the COVID-19 vaccine.

### Analysis plan

Utilizing a rapid analysis approach which produces effective, valid, and timely results, [11, 12] a deductive, rapid analysis tool was created based on the key domains found in the interview guide. The rapid analysis tool was populated independently by members of the analytic team (TWL, SH, YM) for the first three interviews and then reviewed collectively to discuss discrepancies and ensure consistency in the data being recorded. The remaining interviews were then divided amongst the project team, and each lead interviewer subsequently populated the rapid analysis tool for each interview. One team member then conducted a secondary review of the results and discussed discrepancies with team members to further ensure consistency of data recorded. Based on rapid analysis tool results, descriptive statistics (see Table 1) were generated regarding Veteran vaccine hesitancy. Additionally, once the rapid tool was populated, subsequent theme development resulted from team discussions. Ultimately, group discussion and analysis yielded five themes reflective of GeriPACT Veterans attitudes and beliefs about COVID-19 vaccination.

## Results

### GeriPACT Veterans were early adopters of vaccination

Ninety percent of the Veterans who participated in the study reported either receiving the vaccine or being scheduled to receive the vaccine, including 82% receiving the vaccine from VA (See Table 1). Eighty-five percent of the interviewed White Veterans received the vaccine from VA, compared to 82% of the interviewed African American Veterans. Of the two Veterans who had not yet received the vaccine, one was an African American Veteran who had Guillain–Barre’ syndrome and had been advised that he could not take any flu shot. He made the decision to not get the COVID-19 vaccine without advice from his primary care physician. The second was a White Veteran who disclosed that he wanted to let more time pass before getting the vaccine, but declined to be interviewed.

### Vaccinations were facilitated by long-term relationships with VA, which included access to VA resources

The Veterans in our sample had long-term relationships with VA healthcare teams, with a minimum of ten years with VA and two years with the GeriPACT program. Over half our sample was with VA for over 20 years (see Table 2). When asked if they trusted their VA clinical care team, responses such as “If it weren't for my PCP (primary care provider), I don't know if I'd be here right now” (African American Veteran; LR03) were common. This high level of trust for their VA primary care team was complemented by a reported general ease in accessing VA services. And although Veterans described limited social contact and spending the majority of their time in their homes, they were all comfortable describing the distance from their VA facility and their regular routine to attend appointments.

When Veterans were queried about where they had expected to receive the vaccine, everyone in our sample noted they expected to receive the vaccine from VA and noted they had received alerts from VA about how to receive the vaccine.

All Veterans who received the vaccine from VA reported the process to be easy.

“*The VA is so organized. They all knew what they were doing. You got your vaccine and then you were sitting for 15 minutes to be monitored and then if you didn't have any reactions you were let go to go home*.” (White Veteran; LR01)

“*They alerted me, when it was time for my annual physical, that [the COVID-19 vaccine] would be available. They asked me if I wanted to take it, and I said yes. So when I came to take my physical the nurse came and gave me my first shot*.” (African American; SL03)

Although all Veterans who participated in the interviews reported receiving information about how to schedule their vaccine at the VA, few participants reported receiving educational information from VA about the vaccine.

### Trust in VA Team to receive vaccine information

Veterans described being mostly homebound, with 2/3 of our sample living with a spouse, and 1/3 living either alone in a senior living facility or with other family members. Due to being principally homebound, Veterans described the primary sources on the vaccine and vaccination came from TV and on-line news sources or family members. Although the majority of respondents did not report receiving information about the vaccine from their care team, a subgroup did describe proactively reaching out to their clinical team to receive information about the vaccine, and vaccine scheduling, describing them as a trusted resource for healthcare information. One African American Veteran did note an explicit outreach call from his VA primary care team helped convince him to get the vaccine. This Veteran initially did not see a strong need to be an early adopter of the vaccine due to being primarily homebound. He described a long-term relationship with the VA practitioner who called him to discuss the vaccination and stated that the phone call explicitly changed his mind.

### GeriPACT veterans valued vaccine access for caregivers

Veterans frequently described their social spheres during the pandemic to be limited to their immediate household (e.g., their spouse or child). As they typically described themselves as homebound and following pandemic guidelines, they had limited contacts with others except family members who may come into the home, and phone calls with friends. They described rarely leaving their homes, apart from medical appointments.

Two Veterans reported significant frustration at the decision to not provide vaccination opportunities for spouses, as their spouse was their primary caregiver and would be exposed while shopping and engaging in other essential community-based activities.

Veteran: *As old as we are, my wife is my caretaker, I don’t understand that* [why my wife cannot get the vaccine at VA] *at all. That’s not right.*

Interviewer: *If the VA were to call you today and say you’re in line?*

Veteran: *I’d have to tell them no, I refuse it, because they won’t take my wife. I would tell them that.* (White Veteran; SL02)

Veteran*: She* [my wife] *is my home caregiver, but she has not been able to get the vaccine* [from VA]*. What I don’t understand is, if this is a contagious pandemic, if my wife and I are together 22-7, why would there not be a concern if we see each other each day…I don’t understand the reasoning for her not getting the vaccine with me.* (African American Veteran; SL01)

### Reasons for not receiving vaccine from VA

Four primary reasons were identified for not receiving the vaccine at VA, including (1) living in a senior residential facility where the vaccine was administered onsite, (2) not hearing from VA “early enough” and seeking community options, (3) avoiding the technical challenge of registering in VA’s online system, and (4) declining the vaccine because the Veteran’s spouse was ineligible to receive the vaccine at VA.

## Discussion

As the Omicron surge that began in late 2021 demonstrated world-wide, not prioritizing the older old in national vaccinations strategies can result in devastating surges amongst the entire population [13]. Further, recent data shows that even vaccinated and unboosted elderly are dying of COVID at higher rates than unvaccinated adults under the age of 49 [14]. As the largest integrated healthcare system in the United States, and one that serves a disproportionately large percentage of racial and ethnic minorities as well as a large population of individuals with lower socioeconomic means, [15] the VA has an opportunity to serve as an essential access point for COVID-19 vaccinations to the broader population. Although the VA has fully vaccinated more than 2 million Veterans, [16] many more Veterans and their families remain unvaccinated. This study allows VA to better understand barriers to vaccination among some of its most vulnerable Veterans, and the potential role of geriatric primary care programs to optimize their vaccination program.

The three most commonly cited factors influencing vaccine hesitancy in the literature are convenience, confidence, and complacency [14]. These factors have been validated in recent studies of COVID-19 specific hesitancy [17]. In understanding vaccine hesitancy as a continuum, [3] this study illustrates key factors concerning confidence and convenience that emerged to help understand the success of VA in its early vaccination efforts in this GeriPACT sample. The high level of trust reported by both White and African American Veterans enrolled in their VA GeriPACT programs, along with the long-term relationship described with both their current team and the VA, contribute to the VA being a trusted source of healthcare for many patients, bolstering confidence in information received about the vaccine, even when that information is cursory. As important, familiarity with the VA system facilitated easy and rapid registration for and completion of the vaccination process, decreasing barriers around convenience.

The Veteran who presented concern around the vaccine and his Guillain–Barre’ syndrome offers an example of where more interaction with the clinical care team could potentially relieve hesitancy. Concern around Guillain–Barre’ syndrome and the flu vaccine is understandable, and the healthcare community has accordingly dedicated efforts to inform the Guillain Barre community that the COVID vaccine is safe [18].

The primary reasons cited for not receiving the vaccine through VA, or explicitly rejecting early vaccination opportunity, amplify the overwhelming desire to be vaccinated alongside the importance of convenience; technological barriers such as the VA’s online vaccination registration site and the strong desire to be able to get the vaccine with their caregiver, i.e. their spouse. Accessing the internet has been well explored as a barrier to COVID-19 vaccination, [5, 19] and future efforts will require healthcare systems to provide alternate access points for vaccine registration. While initially a limitation, the recent passage of the SAVE LIVES Act [20] now allows for vaccination of Veterans’ caregivers and family members and has tasked the VA with expanding eligibility from 9 million to more than 33 million individuals in the United States [21].

Even in this limited sample, addressing complacency through active outreach efforts by healthcare providers is evident. As noted, the risks to this population exist even though they are often homebound, and herd immunity cannot be achieved without moving many individuals along the continuum to vaccination. The role of healthcare providers in pushing individuals along the vaccine hesitancy continuum has been recognized, [2, 3, 17] and in conjunction with other vaccination promotions, it can serve as a powerful tool.

Our study has limitations. COVID-19 adoption varies by state, and although the project included states representing five distinct regions, acceptance rates may differ in other states. Selection bias may be present since practitioners selected patients for us to interview, and some individuals refused to be interviewed. Although our sample included individuals who declined the vaccine, we did not indicate specific parameters for the practitioners about who to select for the study, so study participants could have been weighted more heavily toward those inclined toward vaccination. Future studies should include individuals from additional states, including rural and other regions where vaccine hesitancy is greater. In addition, VA’s efforts to vaccinate individuals beyond its traditional patient population of eligible Veterans should be examined. Given VA’s nationwide scope and its mandated responsibility to assist the US in times of emergencies and disasters, [22] a successful rollout of efforts to vaccinate these individuals could serve as a model for vaccination efforts when needed for future public health emergencies.

## Conclusion

As the first wave of early adopters of the COVID-19 vaccination effort nears completion, there is a need to target more vaccine hesitant individuals to lower the risk of subsequent waves of infections. Early vaccine adopters valued convenience and did not need extensive educational information from VA to encourage uptake. Creating additional avenues for vaccination registration and including spouses and caregivers in vaccination efforts, with the option of being vaccinated at the same time, could further increase uptake. Although in this limited, small sample we are not yet seeing evidence of vaccine hesitancy within the African American GeriPACT population, further research must explore how racial disparities may influence COVID-19 vaccine hesitancy, and the role of VA in ameliorating these concerns among African Americans and other underserved groups. In addition, the role of previously vaccinated Veterans in influencing vaccine hesitant individuals by addressing the latter’s concerns should be examined.

## Data Availability

The datasets generated and/or analysed during the current study are available from the corresponding author on reasonable request.

## Abbreviations

VA: Veterans Health Administration
PACT: Patient aligned care team
GeriPACT: Geriatric Patient Aligned Care Team
PCMH: Primary care medical home
